# The effects of popular diets on bone health in the past decade: a narrative review

**DOI:** 10.3389/fendo.2023.1287140

**Published:** 2024-03-26

**Authors:** Yue Peng, Zikang Zhong, Cheng Huang, Weiguo Wang

**Affiliations:** ^1^ China Japan Friendship Hospital (Institute of Clinical Medical Sciences), Chinese Academy of Medical Sciences & Peking Union Medical College, Beijing, China; ^2^ Department of Orthopaedic Surgery, China Japan Friendship Hospital, Beijing, China

**Keywords:** ketogenic diet (KD), Mediterranean diet (MD), caloric restriction (CR), high-protein diet, intermittent fasting (IF), bone health

## Abstract

Bone health encompasses not only bone mineral density but also bone architecture and mechanical properties that can impact bone strength. While specific dietary interventions have been proposed to treat various diseases such as obesity and diabetes, their effects on bone health remain unclear. The aim of this review is to examine literature published in the past decade, summarize the effects of currently popular diets on bone health, elucidate underlying mechanisms, and provide solutions to neutralize the side effects. The diets discussed in this review include a ketogenic diet (KD), a Mediterranean diet (MD), caloric restriction (CR), a high-protein diet (HP), and intermittent fasting (IF). Although detrimental effects on bone health have been noticed in the KD and CR diets, it is still controversial, while the MD and HP diets have shown protective effects, and the effects of IF diets are still uncertain. The mechanism of these effects and the attenuation methods have gained attention and have been discussed in recent years: the KD diet interrupts energy balance and calcium metabolism, which reduces bone quality. Ginsenoside-Rb2, metformin, and simvastatin have been shown to attenuate bone loss during KD. The CR diet influences energy imbalance, glucocorticoid levels, and adipose tissue, causing bone loss. Adequate vitamin D and calcium supplementation and exercise training can attenuate these effects. The olive oil in the MD may be an effective component that protects bone health. HP diets also have components that protect bone health, but their mechanism requires further investigation. In IF, animal studies have shown detrimental effects on bone health, while human studies have not. Therefore, the effects of diets on bone health vary accordingly.

## 1 Introduction

Diet is an indispensable component of our daily life, and its impact on the human body has been the subject of extensive research. Over the years, different dietary interventions have been considered as lifestyle interventions that can prevent or treat various diseases such as obesity, cardiovascular disease, epilepsy, and metabolic diseases (1–4). Nutrients participate in every physiological process, regulate metabolism, and play critical roles in each system of the human body, including the skeletal system (5). Various types of diets have different effects on bone health. In this review, we aim to summarize the influences and potential mechanisms of several currently popular diets on bone health, based on both animal and human studies. These diets include the ketogenic diet (KD), the Mediterranean diet (MD), caloric restriction (CR), a high-protein diet (HP), and intermittent fasting (IF). Information about these diets can be found in **Table 1**.

**Table 1:** Characteristics and examples of various types of diets.

Ketogenic diet	Characteristics	High fat intake, moderate protein consumption, and low carbohydrate intake
	Macronutrient ratio	Fat:protein:carbohydrate = 55%–60%:30%–35%:5%–10%
	Type	Classic long-chain triglyceride (LCT) ketogenic diet
		Medium-chain triglyceride (MCT) ketogenic diet
		Modified Atkins diet (MAD)
		Low glycemic index treatment
Mediterranean diet	Characteristics	Plant-focused, healthy fat emphasis
	Common food categories	Vegetable, fruit, bean, whole grain, extra virgin olive oil, nut
Caloric restriction diet	Characteristics	Reduced daily caloric intake, without malnutrition or deprivation of essential nutrients
	Calorie reduction ratio	20%
High-protein diet	Characteristics	High protein focus, elevated consumption, varied sources, balanced nutrition
	Protein intake	More than 25% of calories from protein, over 1.6 g of protein per kilo of body weight
Intermittent fasting	Characteristics	Only eat during a specific time
	Type	Complete alternate-day fasting
		Modified fasting regimens
		Time-restricted feeding
		Religious fasting
		Ramadan fasting

## 2 Method

In this review, we conducted a search of Web of Science’s core database from January 2012 to November 2022, to identify published articles about the effect of different kinds of diets on bone health. The topics were utilized when searching included “ketogenic diet”, “Mediterranean diet”, “caloric restriction”, “high-protein diet”, and “intermittent fasting”, combined with “bone” or “calcium”. All relevant randomized controlled trials (RCTs), observational studies, and reviews were screened and integrated. Case studies, letters, and conference papers or reports were excluded. **Table 2** summarizes most of the cited human studies and **Table 3** summarizes most of the cited animal studies.

**Table 2:** Summary of the animal studies.

Author	Outcome considered and method for evaluating diet and/or bone health parameters	Study type	Model	Findings
Wu et al. (6)	Cancellous and cortical bone architecture	RCT	Forty female C57BL/6J mice randomly divided into four groups: SD+Sham, SD+OVX, KD+Sham, and KD+OVX; fed for 12 weeks.	KD adversely affects both cancellous and cortical bone in long bones. Combining KD and OVX may exacerbate bone loss.
Aikawa et al. (7)	Bone mass, trabecular microstructure and lumbar BMC	RCT	Male C57BL/6 mice randomly divided into four experimental groups: control diet and sedentary, control diet and exercise, LCHF diet and sedentary, and LCHF diet and exercise; fed for 12 weeks.	The LCHF diet impairs bone mass and certain trabecular microstructures in older mice, and reduces the beneficial effects of exercise on lumbar BMC.
Zengin et al. (8)	Trabecular bone volume, serum IGF-I, and the bone formation marker P1NP	RCT	Twelve-week-old male and female Wistar rats randomly divided into three experimental groups: CD, LC-HF-1, and LC-HF-2; fed for 4 weeks.	In male rats, LC-HF diets lead to a reduction in trabecular bone volume, serum IGF-I, and the bone formation marker P1NP, while no such effects are observed in females.
Ding et al. (9)	Bone density and microstructure	RCT	14 male 6-week-old Sprague–Dawley rats randomly divided into two experimental groups: control and KD group; fed for 12 weeks.	The ketogenic diet negatively impacts bone density and microstructure, primarily in appendicular bones, with minimal effects on axial bones like the L4 vertebrae.
Liu et al. (10)	Spinal fusion, microstructures and bone mass	RCT	32 Sprague–Dawley rats randomly divided into two experimental groups: KD and SD; fed for 8 weeks.	KD delayed spinal fusion and decreased bone mass in posterolateral lumbar spinal fusion in rats.
Zhou et al. (11)	BALP, TRACP, OCN, PPAR-γ, cathepsin K, TRAP, bone microstructure, biomechanical properties.	RCT	30 female (aged 8 weeks) C57BL/6J mice randomly divided into three experimental groups: sham, KD, and KD + Rb2; fed for 12 weeks.	Ginsenoside-Rb2 reduced KD-induced bone loss and improved biomechanics, increasing bone volume fraction from 2.3% to 6.0%.
Tagliaferri et al. (12)	Bone density, oxidative stress, inflammation.	RCT	Six-week-old female C57BL/6J mice randomly divided into six experimental groups: 4 OVX and 2 SH; fed for 30 days.	Virgin olive oil with vitamin D3 improved bone density and reduced oxidative stress in OVX mice.
Puel et al. (13)	BMD, spleen weight, plasma fibrinogen levels.	RCT	98 rats randomly divided into seven experimental groups: 20 SH, 26 OVX with standard diet, and 4 additional OVX groups receiving oleuropein at 2.5, 5, 10, or 15 mg/kg body weight; fed for 100 days.	Oleuropein reduced bone loss and improved inflammatory markers in OVX rats at all tested doses except 5 mg/kg BW.
Shen et al. (14)	Body composition, IGF-I, leptin, adiponectin, glutathione peroxidase, TNF-α mRNA, bone volume, BMD, bone strength.	RCT	30 Sprague–Dawley rats divided into HFD, RD, and LFD groups based on weight gain; fed various diets for up to 8 months.	Restricted diet improved body composition but weakened bone structure and strength in obese rats.
Behrendt et al. (15)	Ct.BMD, Tb.BMD, BV/TV, Tb.N.	RCT	Mice divided into CR groups and AL control; fed up to 74 weeks.	Lifelong caloric restriction (CR) worsened cortical bone in young mice but improved trabecular bone in older mice.
Colman et al. (16)	OC, CTX, NTX, PTH, 25(OH)D	RCT	30 male rhesus monkeys fed by CR divided into C and R groups; R group reduced by 100 calories; fed for 3 months.	Long-term caloric restriction (CR) led to a decline in bone mass and density compared to control monkeys, but without pathological osteopenia.
Li et al. (17)	BMAT alterations, BMD	Observational study	BMAd-specific Cre mouse model in which we knocked out adipose triglyceride lipase (ATGL, Pnpla2 gene)	Caloric restriction induced significant increases in genes related to extracellular matrix organization and skeletal development.
Takeda et al. (18)	The BMD of tibia, femoral breaking force and energy	RCT	47 male Wistar rats (5 weeks old) divided into diet and exercise sub-groups; fed for 60 days.	Both inadequate and excessive protein intake can affect bone strength, while a protein intake of approximately 20% promotes bone mass and strength development.
Nebot et al. (19)	TV, BV, BMD	RCT	88 male Sprague–Dawley rats (6 weeks old) divided into 11 groups with SD and HFD diets; fed for 21 weeks.	Caloric restriction resulted in significant alterations in trabecular microstructure, characterized by an increase in trabecular number and a reduction in trabecular spacing, with no changes in bone volume (BV).
Tirapegui et al. (20)	Carcass, proteoglycan synthesis, IGF-I concentration, total tissue RNA, protein concentration and protein synthesis	RCT	16 newly weaned Wistar rats divided into G12 and G26 diet groups; fed for 3 weeks.	Compared to a low-protein diet, a high-protein diet resulted in lower fat mass but showed no significant changes in protein nutritional status.
Kamel et al. (21)	Glucose, insulin, TGs, cholesterol, PTH, OPG, DPD, NTX-1, TRAP-5b, BMD, BMC	RCT	40 male rats divided into control, control+IF, DEX, and DEX+IF groups; treated for 90 days.	IF corrected GIO in rats by inhibiting osteoclastogenesis and PTH secretion and stimulating osteoblast activity.
Kamel et al. (22)	Thyroid abnormality, bone remodeling ability	RCT	8 pregnant Wistar rats divided into fasting and normally fed groups; fed for 21 days after birth.	IF imposed on embryonic rats resulted in a collapse of bone remodeling to some extent.
Shin et al. (23)	BMD	RCT	Female Sprague–Dawley rats divided into four groups: AD-AL, AD-IMF, Non-AD-AL, and Non-AD-IMF; diets for 4 weeks post β-amyloid infusion.	IF exacerbated bone density loss in Alzheimer’s disease-induced estrogen-deficient rats.
Xu et al. (24)	BMD, ALP, TRAP, BMSC	RCT	30 male 6-week-old Sprague–Dawley rats divided into Control, KD, and EODKD groups; fed for 12 weeks.	Compared to KD, EODKD exhibited higher ketone levels but also inhibited the bone resorption process and early bone formation differentiation.

BMD, Bone Mineral Density; BMC, Bone Mineral Content; IGF-I, Insulin-like Growth Factor I; P1NP, Procollagen Type 1 N-Terminal Propeptide; BALP, Bone Alkaline Phosphatase; TRACP, Tartrate-Resistant Acid Phosphatase; OCN, Osteocalcin; PPAR-γ, Peroxisome Proliferator-Activated Receptor Gamma; TRAP, Tartrate-Resistant Acid Phosphatase; Ct.BMD, Cortical Bone Mineral Density; Tb.BMD, Trabecular Bone Mineral Density; BV/TV, Bone Volume per Total Volume; Tb.N, Trabecular Number; OC, Osteocalcin; CTX, C-Terminal Telopeptide; NTX, N-Terminal Telopeptide; PTH, Parathyroid Hormone; 25(OH)D, 25-Hydroxyvitamin D; BMAT, Bone Marrow Adipose Tissue; TV, Total Volume; BV, Bone Volume; ALP, Alkaline Phosphatase; BMSC, Bone Marrow Stromal Cells; TGs, Triglycerides; OPG, Osteoprotegerin; DPD, Deoxypyridinoline; NTX-1, N-Terminal Telopeptide of type I collagen; TRAP-5b, Tartrate-Resistant Acid Phosphatase 5b; SD, Standard Diet; OVX, Ovariectomized; KD, Ketogenic Diet; AL, Ad Libitum; LCHF, Low-Carbohydrate High-Fat; CD, Control Diet; IMF, Intermittent Fasting; SH, Sham-Operated; HFD, High-Fat Diet; RD, Restricted Diet; LFD, Low-Fat Diet; CR, Caloric Restriction; IF, Intermittent Fasting; DEX, Dexamethasone; ICV, Intracerebroventricular; EODKD, Every-Other-Day Ketogenic Diet; RCT, Randomized Controlled Trial; LC-HF-1, “Atkins-Style” Protein-Matched Diet; LC-HF-2, Ketogenic Low-Protein Diet; G12, Libitum Diets Containing 12% Protein; G26, Libitum Diets Containing 26% Protein.

**Table 3:** Summary of the human studies.

Author	Population	Diet	Outcome considered and method for evaluating diet and/or bone health parameters	Findings
Hahn et al. (25)	33 children	KD	Bone mass, Serum 25-OHD levels	KG patients showed vitamin D deficiency and reduced bone mass; Vitamin D supplementation increased KG bone mass by 8.1% in 12 months.
Simm et al. (26)	29 patients	KD	DXA, BMD, BMAD, osteocalcin	Patients on a KD showed a trend towards reduced LS-BMD *Z* scores
Svedlund et al. (27)	39 Children with intractable epilepsy, glucose transporter type 1 deficiency syndrome, or pyruvate dehydrogenase complex deficiency	MAD	Bone mass (total body, lumbar spine, and hip)	MAD has no significant effect on bone mass
Gomez-Arbelaez et al. (28)	20 adult obese patients	KD	BMC and BMD via DXA	KD leaves BMC and BMD statistically unchanged via DXA.
Athinarayanan et al. (29)	349 type 2 diabetes patients	KD	Spine BMD	Diabetes resolution and no adverse effect on bone health were observed in the experiment group.
Bertoli et al. (30)	3 adult patients with GLUT-1 DS	KD	BMD	Long-term KD had no major negative effects on body composition or bone health in adults with GLUT-1 DS.
Vargas-Molina et al. (31)	21 adult resistance-trained women	KD	BMD	KD led to a significant reduction in systolic blood pressure and a small favorable effect on BMD.
Carter et al. (32)	30 obese patients	KD	BSAP, bone turnover ratio, and UNTx	Dieters lost more weight than controls but no significant change in bone turnover markers or ratio was observed.
Heikura et al. (33)	30 world-class race walkers	KD	CTX, OC, and P1NP	Short-term LCHF diet impaired markers of bone modeling/remodeling.
Draaisma et al. (34)	38 epileptic children	KD	Lumbar *Z*-score, BMD	Children on KDT have low normal BMD that may further decrease. Intravenous bisphosphonate therapy showed a statistically significant increase in BMD.
Nestares et al. (35)	59 children with celiac disease (CD), 40 non-celiac children	MD	BMC, bone *Z*-score, and BMD	MD adherence was associated with higher lean mass and bone health in CD children.
Seiquer et al. (36)	20 male adolescents	MD	Calcium absorption and retention	MD led to increased calcium absorption and retention, and decreased urinary calcium excretion.
Julian et al. (37)	492 Spanish adolescents	MD	BMD	Fruits, nuts, cereals, and roots were associated with higher BMC, but significance was lost when adjusted for lean mass and physical activity.
Pérez-Rey et al. (38)	442 premenopausal women	MD	Ad-SOS, BMD	Higher adherence to the MD was positively associated with better bone mass measurements in Spanish premenopausal women.
Cervo et al. (39)	794 community-dwelling men	MD	BMD and risk of incident falls	MD was associated with lower incident fall rates in older men. No association was found between MEDI-LITE scores and BMD or physical function parameters.
Feart et al. (40)	1,482 older French adults	MD	Risk of bone fractures	Higher MeDi adherence was not associated with a decreased risk of fractures in older French persons.
Villareal et al. (41)	218 non-obese, younger adults	CR	BMD, C-telopeptide, TRAP, BSAP	CR led to significant bone loss at crucial sites for osteoporotic fracture due to changes in body composition, hormones, and nutrients.
Tirosh et al. (42)	424 obese and overweight participants	CR	BMD at femoral neck and spine	Weight loss diets had sex-specific effects on BMD: men showed an increase in spine BMD, while women had a decrease in BMD at all sites.
Pop et al. (43)	38 overweight and obese men	CR	Body weight, BMD, BMC, cortical thickness, 25-OHD	CR in overweight and obese men did not decrease BMD or alter bone geometry.
Von Thun et al. (44)	42 postmenopausal women	CR	BMD	People with CR lost BMD at the FN and trochanter after 2 years, irrespective of weight regain or maintenance.
Hinton et al. (45)	40 overweight or obese women	CR	BMD	Hip and lumbar spine BMD decreased with weight loss due to CR and did not recover after weight regain, regardless of exercise.
Armamento-Villareal et al. (46)	107 obese adults	CR	Thigh muscle volume, hip BMD	In the population following CR, thigh muscle mass is related to hip BMD, and a decrease in muscle mass caused by the diet can lead to a decrease in BMD
Antonio et al. (47)	24 exercise-trained women	HP	Whole-body BMD, lumbar BMD, T-scores, lean body mass, and fat mass.	Six months of an HP diet did not affect whole body or lumbar BMD, T-scores, lean body mass, or fat mass.
Lee et al. (48)	12,812 subjects in NHANES	HP	Femoral BMD, T-scores	HP was associated with higher femoral BMD and T-scores in subjects without CKD while CKD patients did not benefit from an HP diet in terms of femoral BMD
Gao et al. (49)	4,447 subjects in NHANES	HP	T-scores, BMD	A high-protein, low-carbohydrate diet may benefit bone health with a significant positive effect on T-score and reduced the risk of low BMD.
Murphy et al. (50)	7 patients with chronic kidney disease and low energy availability	HP	Leptin, IGF-1, P1NP, CTX-I	HP did not mitigate the adverse effects of LEA on bone turnover or leptin levels.
Martens et al. (51)	64 healthy lean midlife/older adults	TRF	Lean mass, BMD	TRF appears to be a feasible and safe dietary intervention for healthy non-obese older adults without negatively impact lean mass, bone density, or nutrient intake.
Clayton et al. (52)	16 lean participants	IF with energy restriction	Serum level of CTX, PINP, PTH	IF with energy restriction does not affect bone metabolism markers like CTX, PINP, and PTH.
Papageorgiou et al. (53)	10 eumenorrheic women	CR	P1NP, CTX, IGF-1, Leptin	Low EA achieved through CR led to a decrease in bone formation but no change in bone resorption.

## 3 Ketogenic diets and bone health

### 3.1 The definition of ketogenic diets

KDs are characterized by a low intake of carbohydrates and a normal to high intake of fat, leading to increased utilization of ketones or fats in the body, similar to changes that occur during periods of starvation. Typically, these diets recommend that only 5% of calories come from carbohydrates, while 75% come from fats and 20% from protein, though the total calorie intake and ratio of energy sources can be adjusted based on individual needs.

### 3.2 Effects of ketogenic diets on bone health

#### 3.2.1 Evidence from animal studies

Most studies have predominantly shown that the KD has an unfavorable effect on bone health. In mice, Wu et al. used the Micro-CT technique and a three-point bending test to assess the bone quality of 8-week-old mice fed a 4:1 KD for 12 weeks. The results indicated that both the cancellous and cortical bone architecture of long bones were compromised (6). A further study on the vertebrae also found a decrease in bone quality (54). Aikawa et al. researched the skeletal systems of aged mice that underwent exercise training and were fed a KD during the experiment, reporting that KD impaired bone mass, trabecular microstructure, and compromised the benefits regarding bone health after exercise (7). Zengin et al. found that a 4-week consumption of an “Atkin-style” KD diet or low protein KD could impair the bone quality of adult male rats. Their femur trabecular bone volume was relatively low, while this effect was not seen in female rats (8). Ding et al. reported that the bone loss was more significant in the appendicular rather than axial bone of rats fed a 3:1 KD (9). Meanwhile, Liu et al. found that a KD can delay the spinal fusion of rats after surgery (10) and confirmed that the microstructures and properties of cancellous bone deteriorated as a result of the interrupted balance of bone resorption and formation (55). Rats fed with KD had significantly lower alkaline phosphatase (ALP) activity and higher tartrate-resistant acid phosphatase (TRAP) activity, and the osteogenic ability of their bone marrow stromal cells was also found to be impaired (56). By measuring TRAP, collagen type I (CoLI), and osteocalcin (OCN) staining, mice fed a KD were found to have upregulated osteoclast activities. When combined with ovariectomy, the osteoblast activities were found to be downregulated (6).

Attention has been drawn to how to relieve the side effect of KD on bone health. Liu et al. found that the bone quality loss induced by KD can be relieved by ginsenoside-Rb2, which inhibits bone resorption and osteogenic differentiation. Metformin can also reduce bone loss by enhancing osteoblast proliferation and inhibiting osteoclast differentiation (11, 57). Zhou et al. demonstrated the protective effect of simvastatin on the bones of mice that were compromised by KD, and the mechanism may be the facilitation of osteoblast differentiation and inhibition of osteoclast differentiation (58). Previous studies have reported that simvastatin can induce the expression of bone morphogenetic protein (BMP)-2, which improves bone formation (59).

#### 3.2.2 Evidence from human studies

##### 3.2.2.1 KD in epileptic children

The alteration of bone health in children treated with KD has been studied since the 1970s. Five epileptic children treated with KD therapy were reported to have disordered mineral metabolism, and their bone mass and serum 25-OHD levels were found to be decreased compared to the normal control (25). In another observational study, researchers investigated the bone health of 29 epileptic children aged 0.5–6.5 years who persisted with a KD for at least 6 months. After measuring with dual-energy x-ray absorptiometry (DXA), they reported a decrease of 0.16 units of bone mineral density *Z* score per year relative to age-matched children (26). In terms of the Modified Atkins Diet (MAD), the intake of protein is not restricted and the KD ratio is 1:1–2:1. A recent study reported that a 24-month MAD did not significantly affect the bone mass and height of children who were diagnosed with intractable epilepsy, glucose transporter type 1 deficiency syndrome, or pyruvate dehydrogenase complex deficiency (27). Most notably, the causes of damaged bone mineral status in epileptic children can also include medication side effects, seizures, and mobile ability (60); thus, more high-level evidence is required to determine the extent of how much KD is to blame for impaired bone growth in epileptic children.

##### 3.2.2.2 KD in adults

The recent applications of the KD in adults were mainly focused on the treatment of metabolic diseases including obesity, diabetes, and glucose transporter 1 deficiency syndrome (GLUT-1 DS). Regarding obesity, a study observed 20 adult obese patients who were treated with the KD for 4 months, and with the measurement of DXA, both their bone mineral content (BMC) and bone mineral density (BMD) were statistically unchanged (28). In terms of diabetes, a study enrolled 262 type2 diabetes patients who were treated with a low-carbonate KD to achieve and sustain nutrition ketosis (blood BHB level of 0.5–3.0 mmol/L). It was found that their spine BMD remained stable from baseline to a 2-year follow-up (29). In regard to GTUT-1 DS, the alteration of bone mass of three adult patients who were treated with normocaloric 3:1 KD for 5 years was observed, and the BMD of all three patients decreased in the first 3 years and remained stable thereafter. At the 5-year follow-up, all patients’ BMDs were in the normal range (30). In a study with healthy participants, 21 adult resistance-trained women were randomly assigned to a non-KD or low-carbohydrate KD group for 8 weeks. The results revealed that the KD group displayed a significant increase in BMD after 8 weeks, while the NKD group showed no significant change. However, no statistical significance was found between the 2 groups (31). In recent years, most studies on adults proved that a KD could improve disease conditions and reduce harm to bone health. However, detrimental effects on bone quality especially in children should be given great consideration. Additionally, because the proportion and type of fat in KD were not always recorded and controlled in current studies, and the duration of KD intervention varied among studies, more high-level studies with standardized study methods and large sample sizes are necessary to reach a final judgement.

**Figure 1:**
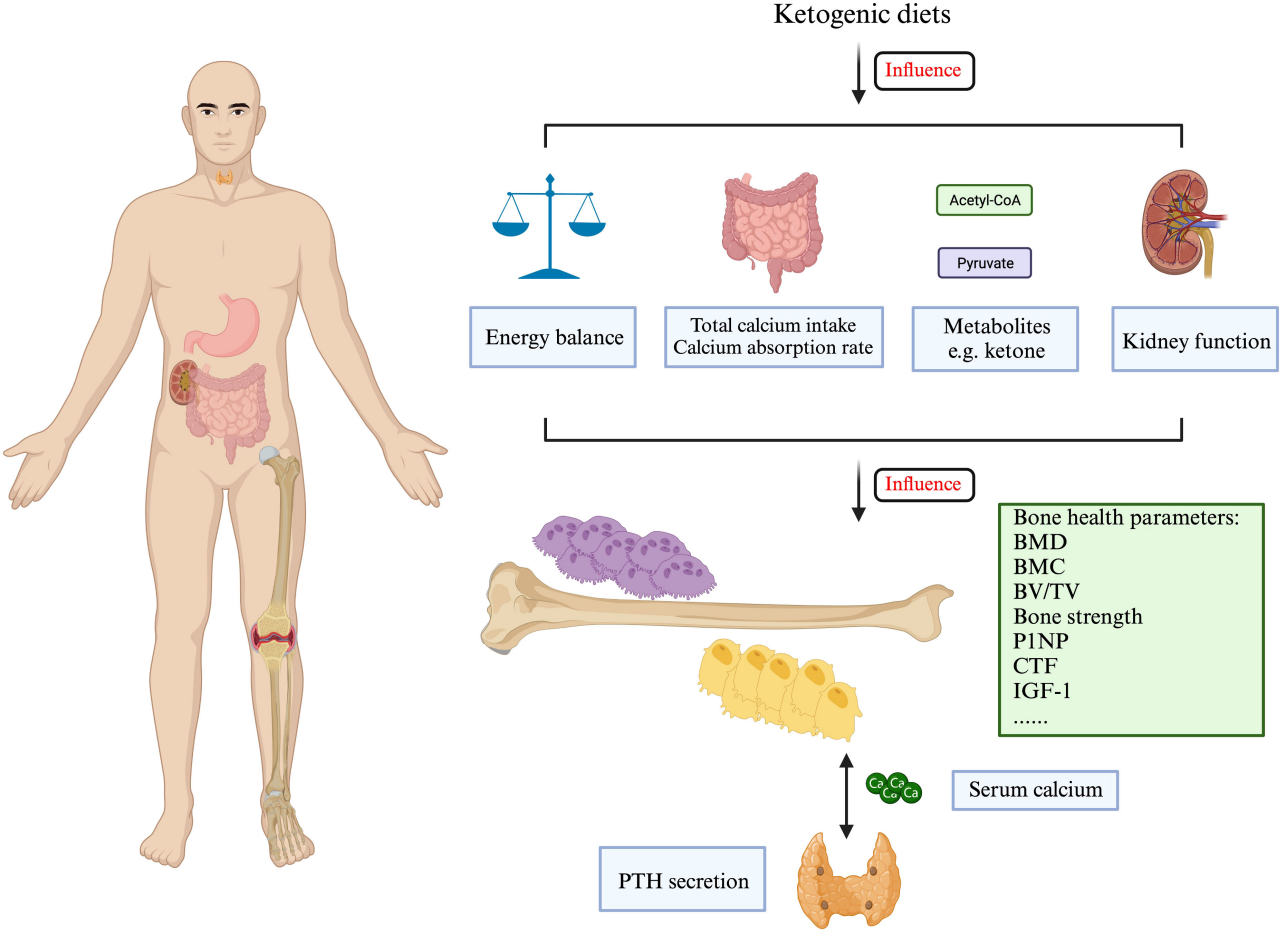
The ketogenic diets may affect bone formation and resorption in multiple ways. Created with BioRender.com.

Current research on the KD indicates that its effects on bone metabolism vary among different age groups and genders. In terms of obese patients, Carter et al. compared 15 obese patients who underwent KD treatment with another 15 matched obese patients without diet intervention for 3 months. No significant difference was found in the comparison of their bone-specific alkaline phosphatase (BSAP) and urinary cross-linked N-telopeptides of type I collagen (UNTx), indicating a negative effect on bone turnover rate in obese patients (32). As to the world-class athletes who underwent a short-term KD for 3.5 weeks, bone resorption markers (cross-linked C-terminal telopeptide of type I collagen, CTX) increased, while the bone formation marker (procollagen 1 N-terminal propeptide, P1NP) decreased (33). Since bone is the major reservoir of calcium, calcium metabolism can provide another perspective on how a KD affects bone metabolism. Current studies have revealed that a KD could decrease calcium digestibility, release calcium from bone to blood, and promote abnormal excretion of calcium. Hawkes et al. observed cases of epileptic children who were treated with KD therapy and then expanded the research into a multi-center study. In general, it was found that children developed hypercalcemia after an average of 2.1 years. Furthermore, moderately elevated urinary calcium excretion, and low levels of serum alkaline phosphatase, PTH, and 1,25-dihydroxyvitamin D were also noticed (**Figure 1**) (61, 62).

Since previous studies have noticed the compromised bone quality of patients treated with KD, research on how to reduce or reverse the side effect of a KD on bone were then conducted, and several antiosteoporosis drugs were reported to be effective. Draaisma et al. conducted a retrospective observational cohort study on epileptic children treated with KD and bisphosphonate for over 6 months; DXA scans were taken to assess the bone mass, and the result showed that bisphosphonate may have a positive effect on the bone mass (34).

## 4 Mediterranean diet and bone health

### 4.1 The definition of a Mediterranean diet

The MD was first defined as being low in saturated fat and high in vegetable oils in the 1960s and has been continuously revised since then. The modern concept of the MD describes it as a diet that includes a high intake of extra virgin olive oil, vegetable, and fruit; a moderate intake of fish and other meat, dairy products, and red wine; and a low intake of eggs and sweets (63). The most recent definition of the MD was released in 2010 by the Mediterranean Diet Foundation (64) (**Table 4**). The MD has been shown to be effective in a variety of diseases, like cardiovascular disease and cancer, as well as in bone health. Its protective effect was due to its antioxidant and anti-inflammatory active molecules such as polyphenols (65, 66).

**Table 4:** The definition of the Mediterranean diet.

Food	Frequency
Sweets	≤2 servings weekly
Potatoes	≤3 servings weekly
Red meat	<2 servings weekly
Processed meat	≤1 servings weekly
Dairy (preferably low fat)	2 servings daily
Olives/nuts/seeds	1–2 servings daily
Olive oil	Every main meal
Fruits	1–2 servings every main meal
Vegetables (variety of color/textures)	≥2 servings every main meal
Bread/pasta/rice/couscous/other cereals	1–2 servings every main meal

Serving sizes specified as 25 g of bread, 100 g of potato, 50–60 g of cooked pasta, 100 g of vegetables, 80 g of apple, 60 g of banana, 100 g of orange, 200 g of melon, 30 g of grapes, 1 cup of milk or yoghurt, 60 g of meat, and 100 g of cooked dry beans.

### 4.2 The Mediterranean diet and bone metabolism

#### 4.2.1 Evidence from animal studies

Until now, only a handful of animal studies have been conducted to reveal the effect of the MD on bone health. Olive oil and vitamin D, which are abundant in the MD, were proven to resist the bone loss induced by estrogen deprivation by regulating the inflammation and oxidative stress status in mice (12). A variety of olive compounds have been studied and proven to have a protective effect on bone. Puel et al. injected ovariectomized rats with osteoporosis (15 mg/kg) with oleuropein, a common component of the MD. After 100 days, the injected rats had a doubled BMD compared with the untreated ovariectomized group. Another study published in 2008 focused on tyrosol and hydroxytyrosol, the main olive oil phenolic compounds. The results showed that ovariectomized rats after 84 days of tyrosol and hydroxytyrosol treatment had higher blood concentrations of osteocalcin and BMD than untreated ovariectomized rats (13, 67).

#### 4.2.2 Evidence from human studies

A study about the MD in children with celiac disease found that it could improve bone health. It was found that both bone mineral content and bone mineral density in these children were significantly increased with high MD adherence than those with low MD adherence. The adherence to the MD was evaluated using the Mediterranean Diet Quality Index in Children and Adolescents (KIDMED) survey. Participants are classified into three categories: (1) high MD adherence (≥8 points), (2) medium MD adherence (4–7 points), and (3) low MD adherence (≤3 points) (35). Another study revealed that compared to a basal diet, male adolescents who adopted an MD as the main meal had a significant improvement in calcium absorption and retention (36). In the meantime, Julian et al. showed that the MD was not associated with BMD (37). Several studies have demonstrated that perimenopausal women with MD had more BMD and trabecular density (38) and less probability of osteoporosis (68–72) than women without. Meanwhile, several studies have indicated that the MD was associated with a reduced risk of fracture, especially in hip fracture (73–75). A case–control study in 2014 that included nearly 700 elderly Chinese persons conducted from 2009 to 2013 with hip fracture showed that a high score in diet-quality scales such as aMed was significantly associated with a decreased risk of hip fractures (76), and a high score in diet-quality scales was often associated with the MD. Other studies revealed that high compliance with the MD was associated with higher BMD and less risk of incident falls (39, 77–79). The protective effect of this diet may be related to the intake of vitamin D3, calcium ions, and the elevated levels of parathyroid hormone in the body (80). However, not all studies found benefits in the MD for bone health. A study conducted from 2000 to 2010 on elderly French persons found no link between the diet and the risk of bone fractures, possibly due to race or environment (40). In the study, individuals with an incident fracture at any of the three sites had a higher mean MeDi score, which assesses MD adherence, at baseline than those who remained free of fracture. Specifically, greater fruit consumption (i.e., >14 servings/week) was significantly associated with a doubled 8-year risk of hip fracture, and a lower intake of dairy products (i.e., <17.0 servings/week in men and <17.9 servings/week in women) was associated with a doubled risk of wrist fracture. It has also been suggested that the olive oil in the MD may reduce the risk of osteoporosis by reducing chronic inflammation (81).

## 5 Caloric restriction diet

### 5.1 The definition of a caloric restriction diet

Caloric restriction (CR) diet is classically defined as a diet with reduced caloric intake, which is approximately 20%–30% below average and does not cause malnutrition during the diet intervention. CR was reported to have the ability not only to reduce weight (82) but also to improve aging-related outcomes (83). However, CR was previously considered as a risk factor for compromising bone quality, and the mechanism behind this phenomenon might be the alteration in bone metabolism, hormones, and weight bearing. Consequently, researchers have tried to introduce a number of remedies to reduce dietary bone damage, including vitamin D intake, high-protein intake, and exercise training (84). Studies in the last decade have gained more results on this topic, which will be reviewed below.

### 5.2 Effects of a caloric restriction diet on bone mass

#### 5.2.1 Evidence from animal studies

Most of the recent animal studies on CR and bone health have once again confirmed the degraded bone mineral condition in rats, mice, and rhesus monkeys. A study on obese female rats implemented a −35% CR diet for 4 months; the BMD, trabecular, and cortical bone volume and bone strength were found to be decreased (14). Another study researched the effect of CR on bone and discovered that the starting age of CR application was found to be crucial to determine its effects. Younger mice showed a more significant loss under CR in terms of cortical bone, cortical BMD, and thickness, compared to senile mice. Long-term CR showed beneficial effects on vertebrae trabecular BMD and BV/TV, which were considered as a reorganization and compensation for the bone loss in cortical bone (15). Issues have also been proposed on whether the decrease of BMD was a pathological process or an adaptation to weight loss. The study on rhesus monkeys indicated that the alteration might be an adaptation process. It was reported that despite the fact that BMD was lower after CR, the alteration of bone turnover markers was not significant; thus, the decreased BMD may be associated with the lower mechanical load generated by a smaller body size, rather than pathological osteopenia (16).

**Figure 2:**
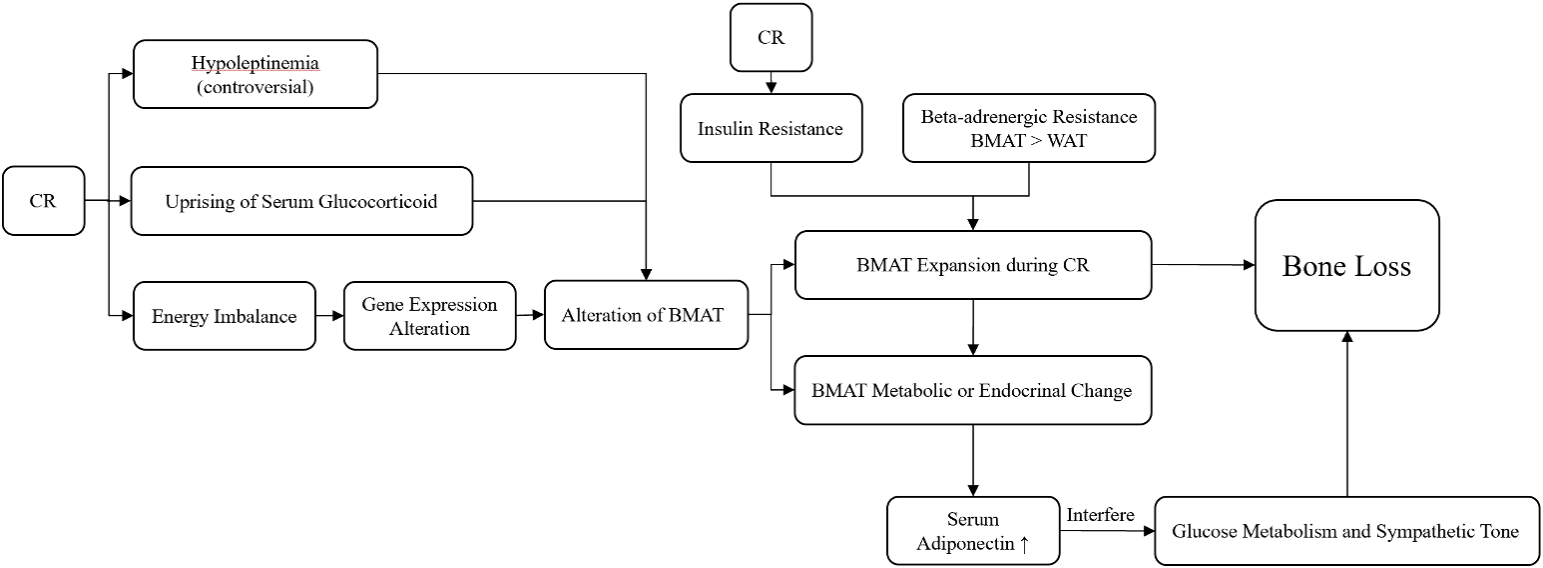
The mechanism of bone–fat interaction during CR.

Regarding the mechanism of how CR affects bone health, recent studies focused most on CR-induced bone marrow adipose tissue (BMAT) alterations. BMAT could respond to CR-induced energy imbalance, cause volume expansion and metabolic or endocrinal change (17), and then cause bone loss. The trigger factors of the alterations in BMAT have been widely studied, and the roles of corticoid have been clarified the most. The uprising of serum glucocorticoid as a result of CR was considered to be relevant (85). The effect of leptin was still unclear, and low serum leptin level was found to be insufficient for BMAT expansion (85). Additionally, despite daily leptin supplementation suppression of BMAT formation in CR mice, it does not attenuate BMD loss or the impairment of bone microstructure; thus, the roles of leptin in bone–fat interaction remain unclear (86). Development of insulin resistance during CR was also found to coincide with BMAT expansion (87). Another study revealed that the preservation of BMAT during CR might be related to its characteristics of beta-adrenergic stimuli resistance compared to white adipose tissue (WAT) (88). Furthermore“, it was proposed that bone–hypothalamus–pituitary–adrenal crosstalk might occur, which may regulate BMAT during CR (89). The alteration of BMAT affected bone health in multiple ways. The expansion of BMAT stored fat, which might take up space in bone marrow, and BMAT released biotic factors that modulated bone turnover. However, whether or not BMAT expansion itself was necessitated in bone loss remains controversial since the amount of expansion might not always be related to the extent of bone loss (87, 90, 91). Adiponectin, secreted by WAT and BMAT, was found to be increased in mice and non-obese adults in CR situations, and overexpression of adiponectin might interfere with glucose metabolism and sympathetic tone, which further affects bone cells and induces bone loss (41, 92). Regarding bone metabolic status, a human study revealed the bone metabolic responses to CR, and based on the evaluation of blood samples, P1NP concentration decreased while CTX concentration remained unchanged, and IGF-1 and leptin levels were decreased, which suggested that CR might induce bone loss and decrease bone formation rather than increase bone resorption (53) (**Figure 2**).

#### 5.2.2 Evidence from human studies

Regarding the determination of whether CR impairs bone quality, while several 2-year studies in non-obese patients, including an RCT study, kept the affirmative opinion (41, 93), other studies found that whether the loss of bone quality existed or not was related to gender differences and the extent of weight loss. An RCT study observed 424 obese and overweight participants, and found decreased BMD in the femoral neck in all patients, while postmenopausal women also showed decreased BMD in the spine. Male patients showed increased BMD in the spine. All participants also underwent regular exercise, which may influence the analysis of the results (42). Another study that investigated 38 men showed that moderate weight loss (−7.9 ± 4.4%) using CR would not decrease BMD at any site or decrease cortical and trabecular bone and geometry (43). Meanwhile, studies have also focused on determining the timing of bone loss during CR. A study on postmenopausal women has shown that the BMD loss did not recover in 2 years after a 6-month CR intervention (44). Moreover, evidence has also shown that bone mineral loss and bone turnover would not recover even after weight regain (45). Furthermore, regarding the assessment tools for the evaluation of BMD in humans who underwent CR diet intervention, aside from the gold standard of DXA, other new predictive tools were investigated. A multiple regression analysis study collected data from 107 obese adults with CR. The researchers selected the changes in thigh muscle volume, lean body mass, osteocalcin, P1NP, CTX, and one-repetition maximum strength as variables. After stepwise multiple linear regression analysis, they demonstrated that changes in thigh muscle volume were positively correlated with changes in hip BMD and were its independent predictor (46).

### 5.3 Methods for attenuating CR-induced bone loss

Considering the potential side effects of CR on bone, researchers have recently focused on interventions that might attenuate such effects, including exercise, vitamin D and calcium supplementation, and other nutrient supplementation.

In terms of exercise, more recent literature confirmed the positive effect of exercise during CR, while a few animal studies disagreed with this opinion. In studies with positive opinions on the subject, RCTs involving overweight/obese adults have found that aerobic training (AT) for 3 months (94) and resistance training (RT) for 5 months (95) were beneficial to weight-bearing bones’ BMD during CR. However, RT might be more effective in bone quality reservation than AT according to the comparison between the BMDs of RT+CR and AT+CR. The potential mechanism needs to be discussed in the future (95). The level of serum sclerostin was found to be higher in participants with exercise training during CR, which might positively influence bone quality (96, 97). In contrast with human studies, animal studies have revealed varying results. Five-month-old female rats that were fed with CR with exercise for 12 weeks have better BMD, BMC, and lean mass compared to rats only fed with CR (98, 99). Another study found that obese rats fed with CR and subjected to exercise for 3 months can attenuate bone volume decrease at the distal femur (100). Nebot et al. proposed a mixed exercise-training protocol with CR and reported that it induced weight loss while preserving bone quality (19). A few studies also pointed out the negative effects of exercise on bone during CR. Hitori et al. conducted a study that randomly divided 14-week-old mature male rats into a control group, a CR group, an exercise group, and a CR+exercise group, and found no significant difference in femur and tibia BMD and trabecular bone volume between groups after 13 weeks (101). They then conducted a similar study on 4-week-old rats; the results indicated that 13-week exercise with CR was detrimental to bone microstructure and strength (102). A more recent study fed 10-week-old rats with CR and subjected them to exercise for 6 weeks. Bone quality was found to be compromised with increased cortical porosity. Exercise also suppressed MAT formation, interrupting its function as an energy supply source to bone formation during CR (103).

In terms of nutrient supplementation, vitamin D and calcium supplementation provided the most significant findings. Over the last 10 years, four RCT studies have revealed different results. A 6-month supplementation of 400 IU vitamin D and 800 mg calcium per day can improve tibial bone properties, which was measured by quantitative CT in young male jockeys who usually undertake CR and high volumes of physical activities (104). Another RCT study observed that a 6-week intake of 1,200 mg calcium and 400 IU vitamin D supplementation in healthy or obese participants during CR could elevate their osteocalcin level and improve insulin sensitivity, which might benefit bone formation (105). Regarding the dose of vitamin D supplementation, an RCT double-blind study found that when calcium intake is 1,200 mg per day, either 10 or 63 μg of vitamin D per day is sufficient to maintain the calcium balance during CR. Calcium balance was evaluated with the parameter of true fractional calcium absorption (TFCA) (106). However, another RCT study revealed that a 12-month vitamin D supplementation (2000 IU/day) did not result in different changes in BMD from placebo in women participating in a weight loss program with CR. It is worth noting that all participants also took part in 225 min/week of aerobic exercise (107). Other nutrient supplementation researched by recent studies included special protein regimens (soy or casein) and Omega-3 polyunsaturated fatty acid (n-3 PUFA) supplementation. However, both of them could not improve bone quality during CR according to the study results (108, 109).

## 6 The effect of high-protein diets on bone health

According to the Recommended Dietary Allowances (RDA) published by the National Research Council (US) in 1989, 0.8 g/kg body weight/day of protein is sufficient for adults, while high-protein (HP) diets refer to diets that contain more than 0.8 g/kg body weight/day of protein. HP diets have gained attention since they have been widely used in the treatment of obesity and diabetes. Furthermore, it is believed that HP diets may improve athletes’ performance and body posture by increasing muscle mass (110–112). However, controversial topics of whether and how HP diets influence bone health still remain. During HP diets, serum IGF-1 and bone matrix collagen synthesis were upregulated, while PTH secretion was downregulated. These factors were beneficial to bone formation. On the other hand, HP diets could also produce more acid during protein metabolism, which could impair bone formation. Overall, the protective effect appears to outweigh the detrimental effect (113).

Animal studies on a moderate-high protein diet showed its positive effect on bone, while even higher protein diets did not seem to further improve bone quality. In a study involving 5-week-old rats fed with different levels of dietary protein and that underwent different levels of exercise, it was found that the BMD of tibia and femoral breaking force were lower in the low-protein-diet group (18). Another study examined 6-week-old obese Zuker rats for 2 months, showing that the combination of an HP diet (25% protein) and exercise enhances the trabecular bone microarchitecture and BMD, while leaving the bone turnover markers unchanged (114). However, a study that tracked 3-week-old rats for 3 weeks did not find any improvement in bone length and bone formation biomarkers in the high-protein group (26% protein) compared with the low-protein group (12% protein) (20). In another study, the HP diet (40% protein) did not improve bone quality more than the moderate-protein diet (20% protein) in rats with high-level exercise (18).

In human studies, no detrimental effects on bone health were found during an HP diet. Researchers compared the HP diet with habitual diets in 24 exercise-trained women for half a year. The results proved that neither the whole-body BMD nor lumbar BMD were significantly different after intervention (47). In another large-sample study, the protein intake of 12,812 subjects with femoral BMD and T scores from the National Health and Nutrition Examination Survey (NHANES) were analyzed. It was demonstrated that BMD and T-scores were positively correlated with the amount of protein intake (48). Another analysis also studied the NHANES database and extracted data from 4,447 subjects. The result showed that diets with a higher percentage of energy from protein were associated with higher T-scores (49). However, the two studies did not record the duration of HP diets. In another aspect, there are studies that have shown that HP diets do not attenuate bone loss in patients with chronic kidney disease and low energy availability (48, 50).

## 7 Intermittent fasting and bone health

Intermittent fasting (IF) is defined as dieting with periodic fasting and non-fasting (115), which includes complete alternate-day fasting, modified fasting regimens, time-restricted feeding (TRF), religious fasting, and Ramadan fasting, thus improving metabolic profiles and reducing the risk of obesity and related diseases (116). Although the intake of calcium was reported to be relatively lower in IF (117), the actual effects of IF on bone health were unclear.

In animal studies, evidence showed the detrimental effects of IF on bone mass and bone remodeling. A study that researched 16 pregnant female rats reported that rats fed with an IF regimen showed a decrease in cortical thickness of the vertebra and the ability of bone remodeling according to the osteoclast count (21). They further observed the offspring of eight pregnant rats fed with IF and found thyroid abnormalities that may be associated with the decrease in bone remodeling ability (22). Another study observed Alzheimer’s disease-induced estrogen-deficient rats, showing that IF could aggravate BMD loss (23). It was also determined that IF may attenuate the detrimental effects of KD on bone. The combination of IF and KD was named Every-other-day ketogenic diet. It was reported that this diet would not impair bone microstructure and strength compared to a normal KD in a rat study (56).

Current human studies showed no detrimental effects in either BMD measurement or bone turnover markers. In terms of IF without energy restriction, a randomized study enrolled 24 healthy lean midlife/older adults, while 10 participants were randomized to stick to their normal feeding pattern for 6 weeks and then transition to a 6-week TRF. The other 14 participants were randomized to stick to the 6-week TRF and then transition to their normal diet pattern; the TRF required participants to consume all meals within a 8-h time window, and the caloric intake was within a regular range to avoid weight loss. In the study results, researchers did not find significant differences between the normal feeding group and the TRF group (51). In terms of IF with energy restriction, another study investigated 16 lean participants for 3 days; on day 1, they consumed a 24-h diet with or without energy restriction (25% of the estimated energy requirement), followed by a standardized breakfast and *ad libitum* lunch and dinner on day 2, and fasting overnight and return on day 3. Their CTX, PINP, and PTH levels were measured on all 3 days, with no differences found between the groups, which indicated that a 24-h severe energy restriction did not affect bone metabolism (52).

## 8 Conclusion

This review presents an overview of the current knowledge on the effects of a KD, an MD, an HP diet, IF, and CR on bone health. We suggest that several problems should be solved first before further addressing the following: (i) Related studies lack standardization of the dietary intervention, which includes the proportion and type of fat in a KD, the energy restriction rate and the nutrition structure of CR, the proportion and resources of protein in HP, the types of IF, the duration of the intervention, and whether calcium supplementation can meet the minimum daily requirement. (ii) The method used for measuring bone quality also lacks standardization, which includes the bone site of measurement and the selection of the assessment tool such as x-ray, computed tomography (CT), or DXA. (iii) Sometimes, studies displayed conflicting results in human and animals; further explanation is needed to address this. (iv) More high-level evidence studies, such as an RCT and meta-analysis of different forms of dietary interventions, should be carried out with a standardized protocol and long-term follow-up. In summary, in a KD and CR, detrimental effects on bone quality were more significant, and attenuation methods were proposed. In contrast, most of the relevant studies on MDs and HP diets showed a positive or non-effective impact on bone health. In IF, recent human studies and animal studies showed different results. Although numerous researchers have been working on this topic for a long period of time, current lines of evidence on human and animal studies were still not sufficient to reach a final solid conclusion.

## Author contributions

YP: Writing – original draft. ZZ: Writing – original draft. CH: Writing – review & editing. WW: Writing – review & editing.
